# Type 2 Diabetes mellitus alters the cargo of (poly)phenol metabolome and the oxidative status in circulating lipoproteins

**DOI:** 10.1016/j.redox.2022.102572

**Published:** 2022-12-05

**Authors:** Ana Reis, Sara Rocha, Irundika HK. Dias, Raquel Costa, Raquel Soares, José Luis Sánchez-Quesada, Antonio Perez, Victor de Freitas

**Affiliations:** a REQUIMTE/LAQV, Department of Chemistry and Biochemistry, Faculty of Sciences, University of Porto, Rua do Campo Alegre 687, 4169-007, Porto, Portugal; b Aston Medical School, Aston University, Birmingham, B4 7ET, UK; c Department of Biomedicine, Faculty of Medicine, University of Porto, Portugal; d CBQF—Centro de Biotecnologia e Química Fina, Laboratório Associado, Escola Superior de Biotecnologia, Universidade Católica Portuguesa, 4169-005, Porto, Portugal; e i3S, Instituto de Investigação e Inovação em Saúde, Universidade do Porto, Portugal; f Cardiovascular Biochemistry, Biomedical Research Institute IIB Sant Pau, Sant Antoni Ma Claret, 167, Barcelona, Spain; g CIBER of Diabetes and Metabolic Diseases (CIBERDEM), 28029, Madrid, Spain; h Endocrinology Department, Hospital de la Santa Creu i Sant Pau, Sant Antoni Ma Claret, 167, Barcelona, Spain

**Keywords:** Flavonoids, Microbial metabolites, Oxidised phosphatidylcholines (oxPC), Endothelial cell, Inflammatory cytokines, Diabetes

## Abstract

The incidence of diabetes on the worldwide population has tripled in the past 5 decades. While drug-based therapies are valuable strategies to treat and ease the socio-economic burden of diabetes, nutritional strategies offer valuable alternatives to prevent and manage diabetes onset and contribute to the sustainability of health budgets. Whilst, intervention studies have shown that (poly)phenol-rich diets improve fasting glucose levels and other blood parameters, very little is known about the distribution of ingested polyphenols in circulation and the impact of diabetes on its cargo.

In this study we investigate the impact of type 2 diabetes on the cargo of plasma (poly)phenols. Our results show that phenolic compounds are heterogeneously distributed in circulation though mainly transported by lipoprotein populations. We also found that diabetes has a marked effect on the phenolic content transported by VLDL resulting in the decrease in the content of flavonoids and consequently a decrease in the antioxidant capacity. In addition to the reduced bioavailability of (poly)phenol metabolites and increase of oxidative status in LDL and HDL populations in diabetes, cell-based assays show that sub-micromolar amounts of microbial (poly)phenol metabolites are able to counteract the pro-inflammatory status in glucose-challenged endothelial cells.

Our findings highlight the relevance of triglyceride-rich lipoproteins in the transport and delivery of bioactive plant-based compounds to the endothelium in T2DM supporting the adoption of nutritional guidelines as an alternative strategy to drug-based therapeutic approaches.

## Highlights

• Phenolic compounds are transported by low- and high molecular weight proteins.
• ApoB-100 containing particles are the main carriers of (poly)phenol metabolites.
• Diabetes markedly reduces the cargo of (poly)phenol metabolites in lipoproteins.
• Diabetes increases the oxidative status of lipoproteins.
• Colonic metabolites dampen inflammation in hyperglycemic endothelial cells.

## Graphical abstract

**Figure:**
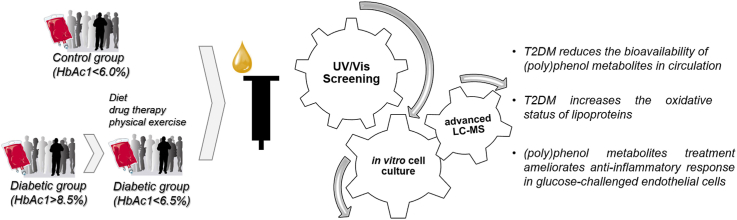

## Abbreviations:

AGE: advanced glycation end-products
BMI: body mass index
CVD: cardiovascular diseases
DHPPA: dihydroxy-phenyl-propanoic acid
DHPV: dihydroxy-phenyl-γ-valerolactone
EDTA: Ethylenediaminetetraacetic acid
EGF: epidermal growth factor
ESI: electrospray ionization
GI: gastro-intestinal tract
glycLDL: glycated low-density lipoprotein
HbA_1c_: glycated hemoglobin
HDL: high-density lipoprotein
HEPES: (4-(2-hydroxyethyl)-1-piperazineethanesulfonic acid) buffer
HMEC-1: human microvascular endothelial cells
hsCRP: high sensitivity C-reactive protein
HUVEC: human umbilical vein endothelial cells
IL-1beta: interleukin 1β
IL-6: interleukin 6
LC-MS: liquid-chromatography coupled with mass spectrometry detection
LDL: low-density lipoprotein
LLE: liquid-liquid extraction
LPO: lipid peroxidation
oxLDL: oxidised low-density lipoproteins
oxPC: oxidised glycerophosphatidylcholines
PCA: protocatechuic acid
PLOOH: phospholipid hydroperoxides
RPMI: cell culture medium
SD: standard deviation
SPE: solid-phase extraction
T2DM: type-2 diabetes mellitus
TAG: triacylglycerides
VLDL: very low-density lipoprotein

## 1 Introduction

The 20th century brought massive changes to people's eating habits. Processed and ready-to-eat foods became routinely consumed resulting in a sharp rise of sugar intake in people's daily diets. The incidence of diabetes on the worldwide population has tripled in the past 5 decades and according to the World Health Organization (WHO) over 537 million people worldwide have been diagnosed with diabetes [1]. Even more alarming is the forecasted number of undiagnosed people and the increasing incidence of diabetes among children and young adults [2]. In an increasingly aged population, the socio-economic impact of diabetes is likely to increase in the near future.

To counteract the deleterious impact of diabetes and attain sustainability of Health systems, the WHO recommends changes to people's eating habits with the inclusion of fresh and unprocessed fruits, vegetables and nuts, typical of Mediterranean- and Nordic-type diets, to prevent and manage diabetes and diabetes associated complications [3].

Epidemiological and intervention studies have shown that the continuous and sustained intake of (poly)phenol-rich foods led to an overall improvement of cardio- and obesity markers [[4], [5], [6]], but also to lower fasting glucose levels thus reducing the risk of type-2 diabetes in healthy individuals [7], improved insulin sensitivity in overweight/obese non-diabetic individuals [8,9] and in diabetic patients [10]. The overall improvement of endothelial and vascular function [5,11] allied with the anti-obesity effect in appetite modulation [[12], [13], [14]], anti-hypercholesterolemic [[15], [16], [17], [18]], anti-hypertensive [16,17,19], and anti-thrombotic effects [[20], [21], [22]] reinforces nutritional strategies as valuable alternatives to minimize endothelial dysfunction and manage the complications associated with diabetes.

Although the anti-diabetic effects associated with the ingestion of (poly)phenol-rich foods may be attributed to the (poly)phenol's ability to inhibit salivary and secreted hydrolytic enzymes responsible for the cleavage and release of glucose during starch digestion by (poly)phenols and consequent glucose uptake [[23], [24], [25], [26], [27]]; upon ingestion dietary (poly)phenols are extensively metabolized to structurally related conjugates (e.g. methyl-, sulphate- and glucuronate derivatives) and to ring-fission (poly)phenol metabolites (e.g. hydroxy-benzoic acids, hippuric acid, phenyl-gamma-valerolactones, hydroxy-phenyl-propanoic acid and many others) [28,29] suggesting that the main health benefits may be due to bioactive metabolites.

In fact, despite the low bioavailability of (poly)phenol metabolites *in vivo* [28,[30], [31], [32]], cell-based studies conducted in the last decade have shown that although metabolization reduces their antioxidant capacity and biological activity, (poly)phenol metabolites are able to reduce glycation of plasma proteins preventing the formation of Amadori and advanced glycation end (AGE) products [[33], [34], [35]], modulate eNOS activity preserving NO bioavailability and vascular function [[36], [37], [38]]; regulate cell-cell adhesion events [21,39]; and are able to modulate the inflammatory response [21,[39], [40], [41], [42], [43], [44], [45]]. Remarkably, in spite of the multifaceted benefits of the (poly)phenol-rich diets very little is known about how these compounds are transported in circulation and how hyperglycemia affects the cargo and the anti-inflammatory activity of (poly)phenol metabolites.

To improve our understanding on these aspects, and better assess on the benefits of polyphenol-rich diets on endothelial health in diabetic patients, this study describes data obtained in lipoprotein populations isolated from normo- and hyperglycemic donors using spectrophotometric and LC-MS targeted approaches. Results were complemented with endothelial cell-based studies on the release of inflammatory cytokines under hyperglycemic conditions.

## 2 Materials and methods

### 2.1 Reagents

Reagents used were of the highest purity commercially available and solvents of MS grade (for additional details see information in Supporting Material).

### 2.2 Lipoprotein isolation

Lipoprotein fractions were isolated by ultracentrifugation protocol as described earlier [46] using pooled plasma samples collected from normoglycemic (n = 15) and diabetic donors (n = 15). Details on study group information are available in Supporting Material. The study was conducted in accordance to the ethical standards of the Declaration of Helsinki and volunteers gave written informed consent prior to participation. The study received approval from the Local Ethics Committee with the reference number IIBSP-REL-2017-27. Anthropometric characteristics, lipid profile, HbA_1c_ and CRP levels of recruited donors are shown in Table 1. Purity of isolated lipoproteins was confirmed by gel electrophoresis in polyacrylamide gel (4–15%, BioRad, Hempstead, UK) stained with colloidal Coomassie solution (for additional details see Supporting Material). Isolated lipoprotein samples were shipped in dry ice and upon arrival immediately stored at −80°C until further use.

**Table 1:** Anthropometric characteristics, plasma lipid profile, HbA1c and hsCRP levels from the subjects included in the study. Data are expressed as Mean ± SD.

	Control group	T2DM-Poor control	T2DM-Good control
Gender (M/F)	7/8	7/8	7/8
Age (years)	49 ± 8	57 ± 9	57 ± 9
BMI (Kg/m^2^)	26.3 ± 1.7	27.1 ± 3.0	25.9 ± 2.1
Total Cholesterol (mmol/L)	5.06 ± 1.06	6.31 ± 1.80*	5.19 ± 1.03^$^
Triglycerides (mmol/L)	0.92 ± 0.36	2.353 ± 1.58*	1.62 ± 1.13*^$^
LDL cholesterol (mmol/L)	3.10 ± 1.16	4.24 ± 1.44*	3.33 ± 0.79^$^
HDL cholesterol (mmol/L)	1.40 ± 0.24	1.05 ± 0.20*	1.09 ± 0.15*
ApoB (g/L)	0.90 ± 0.24	1.39 ± 0.44*	1.06 ± 0.24^$^
hsCRP (mg/L)	1.06 ± 0.73	4.75 ± 4.95*	3.51 ± 3.29*
HbA1c (%)	5.19 ± 0.20	11.22 ± 1.70*	6.24 ± 0.79*^$^

*P < 0.05 vs control group.

^$^ P < 0.05 vs T2DM-PC.

### 2.3 Extraction of (poly)phenolic metabolites by solid-phase extraction (SPE) and characterization by spectrophotometric methods

Extraction of (poly)phenol metabolites from isolated lipoprotein samples was carried out by solid-phase extraction (SPE) using Hybrid SPE-Phospholipid cartridges (Supelco, Saint Louis, USA) according to the manufacturer's instructions assisted by a HyperSep™ Glass Block Manifold. Lipoprotein extracts (n = 3) were characterized in 96-well microplate by spectrophotometric assays. Total phenol content of extracts was measured by the Folin-Ciocalteu assay Singleton and Rossi (1965) using gallic acid as the standard. Total phenol content was expressed in μmol of gallic acid equivalents/mg of protein (μmol GAE/mg protein). Total flavonoid content was measured as described earlier [46] using catechin as the standard. The concentration of each sample was measured (triplicate) against the calibration curve and results expressed as μmol of catechin equivalents/mg of protein (μmol CE/mg of protein). The ferric reducing antioxidant power (FRAP) of lipoprotein extracts was measured according to the method of Benzie and Strain (1996) as described earlier [46] using ferrous sulphate (FeSO_4_) solution as the standard. The concentration in each well was calculated against the calibration curve and results (n = 3) expressed as μmol of Fe^2+^/mg of protein. The anti-radical ability (DPPH assay) was carried out as described previously [46] using Trolox as the standard and results expressed as μmol of Trolox equivalents (TE)/mg protein.

### 2.4 Quantification of (poly)phenol metabolites in extracts by reverse-phase liquid chromatography coupled to mass spectrometry (LC-MS)

Dried lipoprotein extracts under vacuum at 30°C (CentriVap Benchtop Vacuum Concentrator, Labconco) were resuspended in 100 μL of 10% ACN (v/v, eluent A) and injected in a reverse-phase column Hypersil GOLD™ VANQUISH™ C18 UHPLC Column (150 mm × 2.1 mm, 1.9 μm, Thermo Fisher, USA) coupled to an ion trap Finnigan LCQ DECA XP MAX equipped with an electrospray ionization (ESI) interface. For details of LC elution conditions and MS acquisition parameters (Supplementary Table 1), see Supporting Material. The content of DHPV, PCA, DHPP and hippuric acid and their sulphate and glucuronic acid conjugates was expressed as pmol/mg protein.

### 2.5 Quantification of phosphatidylcholine oxidation products (OxPC) in extracts by targeted LC-MS approach

Oxidation products of glycerophosphatidylcholine lipids (oxPC) were quantified through targeted LC-MS approach described earlier [47] in lipoprotein extracts prepared from 25 μL of lipoprotein. Raw data analyses were performed in Analyst software (version 1.6.2). Concentration of short-chain OxPC in extracts (n = 3) was calculated against standard curves and data expressed as μmol/L.

### 2.6 Cell culture of human microvascular endothelial cells

Human microvascular endothelial cells (HMEC-1, ATCC) were cultured in RPMI 1640 medium supplemented with 10% FBS, 1% penicillin/streptomycin, 1.176 g/L sodium bicarbonate, 4.76 g/L HEPES, 10 g/mL EGF and 1 mg/L hydrocortisone (purity >98%) and maintained at 37°C in a humidified 5% CO_2_ atmosphere. All experiments were performed between cell passages 6 and 11. Treatment of HMEC-1 cells was done in serum-free cell medium with 5.5 mM glucose, to mimic the normoglycemic condition, or 30 mM glucose concentration, to mimic diabetic condition for 24 h followed by the addition of the (poly)phenol metabolites (0.1–5 μM, in 0.1% ethanol) to the medium and left to incubate for 6 h (triplicates). Cell supernatants were collected and stored at −20°C until further analysis.

### 2.7 Cell viability assay

HMEC-1 cells were sub-cultured in 96-well plates at density of 2 × 10^5^ cells/mL of medium and treated as described in section 2.6. After incubation with (poly)phenol metabolites, medium was replaced with medium containing glucose and tetrazolium salt (MTS). After 1 h in the dark, the absorbance was measured (λ = 492 nm) and results expressed as percentage change relative to control (medium with 5.5 mM glucose).

### 2.8 Enzyme-linked immunosorbent assay for inflammatory markers IL-6 and IL-1beta

Quantification of cytokines (IL-6 and IL-1β) in HMEC supernatant was achieved by ELISA protocols (Sigma-Aldrich, Germany) according to the manufacturer's instructions. HMEC-1 were seeded and allowed to grow to 24 × 10^4^ cells/well in 24-well plates and treated as described in section 2.6. The control used was 0.1% (v/v) ethanol. The results are expressed as pg/mL.

### 2.9 Statistical analysis

Statistical analysis was done on mean ± SD values using the one-way ANOVA and Bonferroni test in GraphPad Prism version 8.4.3 (GraphPad Software, USA). Significance was determined at p < 0.05.

## 3 Results

### 3.1 Phenolic compounds are heterogeneously distributed across lipoproteins

To improve our understanding on the transport of (poly)phenol metabolites in circulation, lipoprotein and lipoprotein-depleted fractions isolated from pooled plasma collected from fasting normo- and hyperglycemic donors were analysed for their total phenolic content. Preliminary data obtained on the extraction performance of (poly)phenol metabolites from normoglycemic plasma sample through liquid-liquid and solid-phase extraction protocols (Supplementary Fig. 1) revealed that Oasis HLB SPE cartridges extracted higher amounts of phenolic compounds when compared to SPE Hybrid-PL cartridges and liquid-liquid extraction (LLE) protocols. Results obtained also showed that Oasis HLB SPE cartridges extracted plasma lipids that are likely to affect column performance and lead to ion suppression effects during metabolite MS detection. As PL Hybrid SPE cartridges displayed the highest phenolic/phospholipid ratio (inset in Supplementary Fig. 1), extracts of lipoprotein and lipoprotein-depleted fractions were subsequently prepared using PL Hybrid SPE.

**Fig. 1:**
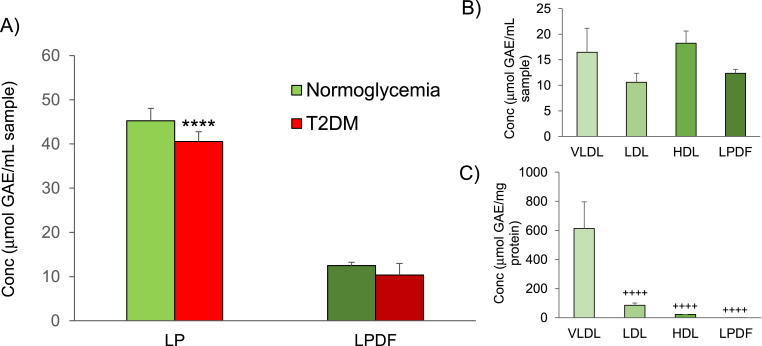
Total phenolic content in A) lipoprotein (LP) and lipoprotein-depleted fraction (LPDF) in normoglycemia and hyperglycemia; B) Phenolic content in lipoprotein populations in normoglycemia showing absolute values (μmol GAE/mL sample) and (C) values normalised to protein content (μmol GAE/mg protein). Values are expressed as mean ± SD (n = 3). (****p < 0.0001 compared to normoglycemia; ++++p < 0.0001 relative to VLDL).

The phenolic content of lipoprotein (VLDL, LDL and HDL) extracts from normoglycemic donors is higher than that of lipoprotein-depleted fraction (Fig. 1A) suggesting that phenolic compounds are transported in circulation by both high and low molecular weight plasma proteins (apolipoproteins and albumin). Analysis of extracts from diabetic donors shows that hyperglycemia induces a slight decrease in the amount of phenolic compounds in both fractions (Fig. 1A).

Detailed analysis of lipoprotein populations in normoglycemia shows that phenolic compounds are distributed across VLDL, LDL and HDL lipoproteins (Fig. 1B). However, as lipoprotein populations have very different chemical composition and physical properties (size and density), absolute values depicted in Fig. 1B may simply reflect the higher number of smaller HDL particles per volume (mL) when compared to the lower number of VLDL particles. In an attempt to take into account the large differences of lipoproteins particle size, phenolic content was normalised to protein content. Normalization of values shows that phenolic compounds are heterogeneously distributed across lipoprotein populations (Fig. 1C) with VLDL as the main carriers of phenolic compounds, suggesting that these compounds are predominantly transported by high molecular weight apoproteins (ApoB-100).

### 3.2 T2DM reduces the bioavailability of (poly)phenol compounds in circulation

**Fig. 2:**
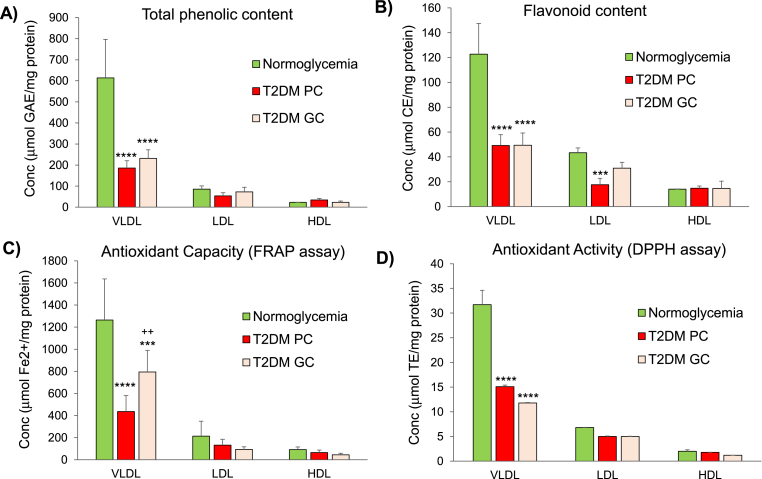
Profile of total phenolics, flavonoid content, antioxidant capacity in lipoprotein populations in normoglycemia and type 2 diabetes (T2DM) with poor control (PC, HbA1c>8.5%) and T2DM with good control (GC, HbA1c<6.5%). Values are expressed as mean ± SD (n = 3) (**p < 0.01, ***p < 0.001, ****p < 0.0001 compared to normoglycemia; ++p < 0.01 relative to T2DM PC).

Purified lipoprotein populations (Supplementary Fig. 2) from age- and gender matched normoglycemic group (HbA1c<6.0%), from diabetic group with poor control (HbA1c>8.5%) and from diabetic group after glycemic control improvement through diet and drug therapy (HbA1c<6.5%) were characterized according to their chemical composition (Table 2) and to their phenolic and flavonoid content, and antioxidant activity (Fig. 2). As can be seen by spectrophotometric characterisation of lipoprotein extracts (Fig. 2) shows that T2DM induces a marked decrease (70%) in the content of phenolic compounds transported by VLDL as well as in LDL suggesting that hyperglycemia reduces the bioavailability of (poly)phenol metabolites in circulation (Fig. 2A). The levels of phenolic compounds are slightly restored in patients undergoing diet and drug therapy. The marked decrease in the content of phenolic compounds with T2DM may be related to the decrease in the content of flavonoids transported by VLDL (Fig. 2B). In consequence of the diminished content of phenolic and flavonoid compounds in T2DM donors in comparison to normoglycemic donors, the antioxidant activity is greatly diminished (Fig. 2C and D). Data also reveals that improvement of glycemic control by complementary diet and drug-based therapies slightly improves the antioxidant capacity of VLDL lipoproteins (Fig. 2C).

**Table 2:** Biochemical composition of lipoproteins isolated from pooled plasma of healthy (normoglycemic group) and diabetic donors with good glycemic control (GC, HbA1c<6.5%) or poor glycemic control (PC, HbA1c>8.5%) (see Section 2.2 for details). The values shown for lipids are expressed in mM and for proteins expressed in g/L. Values in curved brackets express % of total lipoprotein mass with an accuracy of ±5%.

Lipoproteins	Study Group	Chemical composition					
		Total cholesterol	Triglycerides	Phospholipids	Free Cholesterol	Esterified Cholesterol	ApoB-100
**VLDL**	Normoglycemia	0.58 (23.6)	0.44 (41.5)	0.25 (22.1)	0.16 (6.2)	0.42 (17.3)	0.16 (12.8)
	T2DM (PC, HbA1c>8.5%)	2.90 (21.0)	3.01 (49.9)	1.35 (19.6)	1.27 (9.3)	1.63 (11.7)	0.51 (9.6)
	T2DM (GC, HbA1c<6.5%)	3.36 (20.9)	3.45 (49.0)	1.58 (20.0)	1.48 (9.2)	1.88 (11.7)	0.62 (10.0)
**LDL**	Normoglycemia	3.32 (38.7)	0.29 (7.8)	1.12 (26.6)	0.88 (10.3)	2.44 (28.4)	0.89 (26.9)
	T2DM (PC, HbA1c>8.5%)	11.2 (38.6)	0.96 (7.5)	3.72 (25.7)	2.94 (10.1)	8.26 (28.5)	3.15 (28.2)
	T2DM (GC, HbA1c<6.5%)	13.1 (38.2)	1.15 (7.6)	4.33 (25.3)	3.30 (9.6)	9.80 (28.7)	3.80 (28.8)
**HDL**	Normoglycemia	1.28 (15.9)	0.13 (3.6)	1.12 (28.0)	0.20 (2.5)	1.08 (13.4)	1.23 (39.9)
	T2DM (PC, HbA1c>8.5%)	2.91 (12.7)	0.34 (3.5)	2.54 (23.6)	0.64 (2.8)	2.27 (10.0)	4.31 (49.5)
	T2DM (GC, HbA1c<6.5%)	2.11 (11.9)	0.28 (3.5)	2.01 (23.0)	0.48 (2.8)	1.63 (9.1)	3.13 (46.9)

As the Folin-Ciocalteu spectrophotometric method is not specific for (poly)phenol compounds [48] quantification of (poly)phenol metabolites in lipoprotein extracts was also conducted by targeted LC-MS approaches. The panel of (poly)phenol metabolites quantified focused on microbial metabolites formed by ring-fission of flavonoids such as hydroxy-benzoic acids, hippuric acid, phenyl-γ-valerolactones and phenyl-propanoic acid, rather than the structurally-related conjugates (e.g. sulphates and glucuronates), as blood samples were collected after overnight fasting. Concentration of (poly)phenol metabolites in lipoproteins for the 3 donor groups (Supplementary Table 2) ranged between 400 nM and 21 μM with di-hydroxy-phenyl-γ-valerolactones as the most abundant metabolite, accounting for more than 90% of the total of metabolites quantified. Values of individual (poly)phenol metabolites (pmol/mg protein) were summed and estimates of the total (poly)phenol metabolites across lipoproteins are depicted in Supplementary Fig. 3. Results obtained from targeted LC-MS approach corroborate our previous spectrophotometric data (Fig. 2A) showing that (poly)phenol metabolites are heterogeneously distributed across lipoprotein populations and that T2DM greatly reduces the amount of (poly)phenol metabolites available in circulation.

### 3.3 T2DM increases the oxidative status of circulating LDL and HDL

**Fig. 3:**
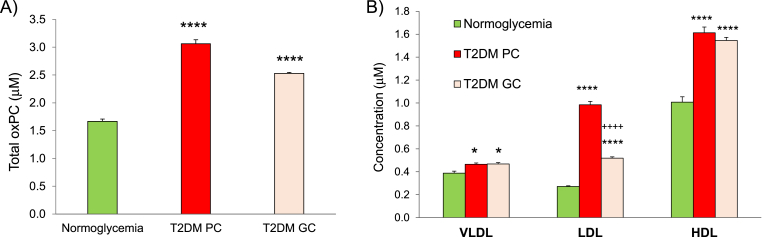
Estimated levels of total oxPC in lipoproteins from A) normoglycemic donors (control), type 2 diabetes (T2DM) with poor control (PC, HbA1c>8.5%) and T2DM with good control (GC, HbA1c<6.5%) and B) in isolated lipoprotein populations. Values are expressed as mean ± SD (n = 3). Significance test *p < 0.1, ****p < 0.0001 relative to NG; ++++p < 0.0001 relative to T2DM PC.

In addition to reduced bioavailability of (poly)phenol metabolites in circulation, T2DM also increased the oxidative status of all lipoprotein populations. As shown by our data on the content of 8 fragmented oxidised phosphatidylcholines (oxPC) through targeted LC-(MRM)-MS approach, T2DM led to a 2-fold increase on the levels of total oxPC transported by lipoproteins (Fig. 3A).

Results obtained in individual lipoprotein populations from normoglycemic donors show that oxPC are distributed across all lipoprotein populations with predominance in HDL particles (Fig. 3B). The levels of oxPC are raised in all lipoprotein populations in T2DM with the highest increase for LDL (3-fold) and a 30% increase for HDL. As shown by our data, diet and drug-based therapies had a marked effect (48%) in restoring oxPC of LDL in diabetic donors and a less pronounced effect on HDL (Fig. 3B).

### 3.4 Microbial (poly)phenol metabolites decrease the release of IL-6 in glucose-challenged endothelial cells

(Poly)phenol metabolites quantified in lipoproteins (Supplementary Fig. 3) were evaluated towards the inflammatory response of endothelial HMEC-1 cells exposed to hyperglycemia by measuring the release of IL-6 and IL-1β into the cell medium after treatment. To ensure that (poly)phenol metabolites treatment had no influence on HMEC-1 cell viability, cells were treated with protocatechuic acid (PCA), dihydroxy-phenyl-valerolactone (DHPV) and dihydroxy-propanoic acid (DHPPA) for 6 h under normoglycemia (5.5 mM glucose) and hyperglycemia (30 mM glucose). Results obtained (shown in Supplementary Fig. 4) reveal that treatment of HMEC-1 cells with the microbial (poly)phenol metabolites had no significant impact on cell viability after 6 h of incubation time.

**Fig. 4:**
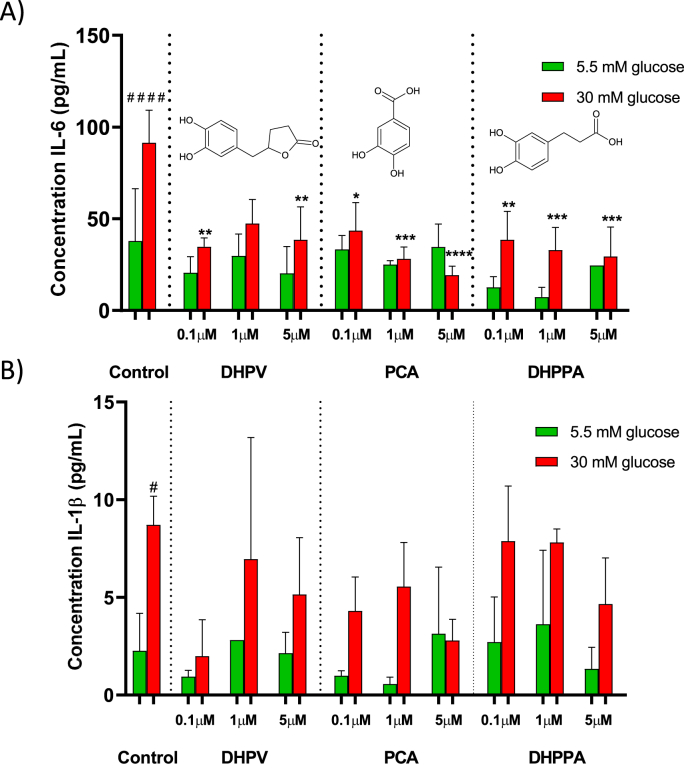
Effect of DHPV, PCA and DHPPA treatment in the release of IL-6 (A) and IL-1β (B) in HMEC-1 cells exposed to normo- and hyperglycaemia conditions (24 h) prior to (poly)phenol metabolite treatment (6 h). Data are expressed as Mean ± SD (n = 3). The control was carried out with 0.1% ethanol (v/v). Significant difference relative to 5.5 mM glucose control conditions are expressed as ####p < 0.0001 and #p < 0.05; Significant difference relative to 30 mM glucose control conditions are expressed as ****p < 0.0001; ***p < 0.001; **p < 0.01; and *p < 0.05.

Exposure of HMEC-1 cells to hyperglycemia conditions induces a significant increase in the release of inflammatory cytokines IL-6 (90.2 ± 7.80 pg IL-6/mL) and IL-1β (8.71 ± 1.48 pg IL-1β/mL) in comparison to the normoglycemia conditions (33.9 ± 11.9 pg IL-6/mL and 2.26 ± 0.86 pg IL-1β/mL), as shown in Fig. 4A and B. Treatment of HMEC-1 cells (grown under hyperglycemic conditions) with colonic (poly)phenol metabolite at 0.1, 1 and 5 μM for 6 h led to an overall decrease in the release of IL-6 cytokines (up to 40%) to the cell culture medium (Fig. 4A) suggesting an anti-inflammatory effect. Even though the panel of (poly)phenol metabolites tested is small and not representative of the whole panel of (poly)phenol metabolites in circulation, results obtained for IL-6 (Fig. 4A) suggest that the anti-inflammatory effect is concentration-dependent and this may be related to the structural details of colonic (poly)phenol metabolites namely the presence of phenyl ring. Treatment with (poly)phenol metabolites showed no detectable concentration effect on the IL-1β release for the different concentrations of DHPV, PCA and DHPPA between the 30 and 5 mM glucose conditions and hence, with the exception of control conditions, no statistically significant effect was observed after 6 h of incubation (Fig. 4B). Nevertheless, this is the first study investigating the effect of microbial (poly)phenol metabolites at physiological relevant conditions in glucose-challenged EC.

## 4 Discussion

Recent epidemiological and intervention studies highlighted the health benefits of continuous and sustained intake of (poly)phenol-rich foods have in the management of T2DM [8,11,[49], [50], [51]] offering valuable and sustainable alternatives on the treatment and management of T2DM in an increasingly aged society. Nevertheless, in spite of the extensive work conducted in the last decade on the absorption, distribution, metabolization and excretion of human (poly)phenol metabolome [28,52,53], our data provides an improved knowledge on how these bioactive compounds are transported in circulation and the effect of T2DM on their cargo.

### 4.1 Triglyceride-rich particles are the main carriers of (poly)phenol metabolites

Our data shows that phenolic compounds are transported by high- and low-molecular weight plasma proteins (Fig. 1B), however in view of the large differences of particle size between VLDL, LDL and HDL [54,55] and the changes to size reported in T2DM [56], values were normalised to the content of protein. Normalised data reveals that phenolic compounds are mainly transported by VLDL particles (Fig. 1C) well in agreement with previous work investigating the distribution of resveratrol in plasma proteins [57]. Previous work by Belguendouz and colleagues (1998) showed that although quantitively resveratrol was associated with the non-lipoprotein fraction, they noticed that after fractionation of lipoproteins resveratrol was distributed according to the lipid content HDL < LDL < VLDL [57].

Our work also shows for the first time the dramatic impact T2DM has on the cargo of phenolic compounds and of flavonoids transported by lipoproteins leading to a major decrease in their content (Fig. 2A and B) thus reducing the bioavailability of these bioactive compounds in circulation. Concomitantly, the decrease of antioxidant capacity in lipoproteins in T2DM is observed (Fig. 2C and D) and in line with others reporting the reduced antioxidant capacity in plasma samples from diabetic patients and slightly restored after improved glycemic control [58].

Data from targeted LC-(SIM)-MS approach (Supplementary Fig. 3) confirms spectrophotometric data (Fig. 2A) showing that (poly)phenol metabolites are heterogeneously distributed across lipoprotein populations and that T2DM significantly reduces the cargo of (poly)phenol metabolites in lipoproteins, particularly in VLDL. The reduced bioavailability of (poly)phenol metabolites observed in T2DM may be due to a combined effect of factors, namely by the impairment of metabolising pathways in PC control group and to a drug-mediated effect in GC group considering that drug-therapies are known to alter lipoprotein's lipidome [59]. Although the (poly)phenol metabolome in isolated lipoproteins remains poorly studied, levels of individual metabolites (PCA, DHPP and hippuric acid) reported in this study are within the range of values reported earlier for tyrosol-based metabolites (240 pmol/mg ApoB-100 protein) in LDL of healthy women [60]. Overall values of (poly)phenol metabolites ranged between 100 nM and 21 μM (Supplementary Table 2) which are within plasma values reported in healthy individuals following intermittent [31,32,61] and sustained (poly)phenol-rich diets [[62], [63], [64]].

Nevertheless, it should be bear in mind that the content of (poly)phenol metabolites is higher than that here reported (Supplementary Fig. 3), as the panel of 12 metabolites here included is not representative of the whole (poly)phenol metabolome [32,63,65,66]. It should also be taken into account, that Hybrid PL SPE cartridges used in the extraction step displayed higher phenolic-to-phospholipid ratio when compared to LLE and Oasis HLB protocols (inset in Supplementary Fig. 1) though moderate performance in the extraction of phenolic compounds (Supplementary Fig. 1) when compared to Oasis HLB cartridges. This is in agreement with others reporting the use of acidic acetonitrile combined with SPE removal of phospholipids as the choice for nutrimetabolomic studies [67,68] even though Oasis HLB is the most popular approach for biological samples [31,32,60,63].

### 4.2 T2DM induces changes to lipoproteins’ chemical composition likely to impact the particles biophysical properties

Previous studies have shown that (poly)phenols interact with plasma proteins through hydrophobic interactions with high binding affinities [[69], [70], [71]]. Results from this study show that triglyceride-rich VLDL lipoproteins containing low protein content exhibit higher cargo of (poly)phenol metabolites than LDL or HDL particles with higher protein content (Supplementary Fig. 3). Considering that VLDL and LDL are both ApoB-100 containing lipoproteins, our results show that (poly)phenol metabolites have a higher affinity for more fluid triglyceride-rich VLDL particles than the more rigid cholesterol-rich LDL particles suggesting that adsorption of (poly)phenol metabolites may be mediated by the lipoprotein's lipid environment. In T2DM the same trend is observed though, hyperglycemia led to a marked decrease (>79%) in the cargo of (poly)phenol metabolites transported by all lipoproteins (Supplementary Fig. 3). As the reduction of (poly)phenol cargo is within the same order of magnitude for all lipoprotein populations, the changes observed are likely consequence of the T2DM-induced changes to the particle's lipid environment (Table 2). Interestingly, diabetic donors with GC following hypoglycemic therapy display a slight improvement of both blood lipids (Table 2) and also on the phenolic content (Fig. 2). Hence, the reduced bioavailability of (poly)phenol metabolites in T2DM (Supplementary Fig. 3) suggests a strong association to the particle's lipid environment.

These changes together with the increase in oxPC content in LDL and HDL (Fig. 3B) are surely to impact the biophysical properties of lipoproteins. In fact, previous studies have shown that the incorporation of triglycerides into lipoproteins resulted in increase of particle fluidity [72] with decrease of VLDL stability [73] whereas the incorporation of cholesterol and oxPC resulted in increased stiffness (decreased membrane fluidity) in model particles (liposomes) [[74], [75], [76]] and in endothelial cells [[77], [78], [79]]. Although, it is widely recognized that surface (poly)phenols together with deeply buried lipophilic antioxidants provide antioxidant protection to lipoproteins [[80], [81], [82], [83]] more recent studies have shown that dietary (poly)phenols fluidify cholesterol-rich environments [84,85]. In view of this and the impact of lipid changes in modulating the fluidity in membranes and lipoproteins [78,[86], [87], [88], [89], [90]], the reduced bioavailability of (poly)phenol metabolites in T2DM may turn fluid triglyceride-rich VLDL even more fluid, and prevent the binding of VLDL to endothelial lipoprotein lipase (LPL) to release TAG [91] leading to the accumulation of circulating TG-rich particles in T2DM patients.

### 4.3 T2DM increases the oxidative status of atherogenic LDL

Raised levels of glucose in circulation have a marked impact on endothelial cell energetics [92] with impairment of endogenous antioxidant systems, and exacerbated production of reactive oxygen species (oxidative status) thus increasing the susceptibility of lipoproteins to oxidative modification [93] as was confirmed in this study (Fig. 3A). Additionally, as can be seen from the particle's characteristics (Table 2) data shows that the protein content in LDL from T2DM is higher suggesting that the LDL size is decreased when compared to control subjects. Previous studies have shown the prevalence of smaller LDL in diabetic patients [94,95] making it more oxidizable [96].

Curiously, although others have previously reported the increased content of plasma oxPC in inflammatory-related conditions [47,97,98], our results show for the first time how oxPC are distributed across lipoproteins in normo- and hyperglycemia (Fig. 3B). The distribution profile of oxPC reveals that HDL exhibit the highest content of oxPC well in agreement with the uneven distribution of phospholipid hydroperoxides (PLOOH) reported between LDL and HDL in fasting healthy donors [99]. The predominance of oxPC in HDL particles is likely due to the low content of lipophilic antioxidants (alpha-tocopherol, γ-tocopherol, ubiquinone Q-10, beta-carotene and others) in HDL particles [[100], [101], [102]], nevertheless it should also be considered the high affinity of apoA-I towards these oxidised lipids conferring HDL with an antioxidant role minimising the widespread oxidation of LDL [103].

Though co-localization of oxPC at the surface of lipoproteins remains vague [104] it is widely accepted that fragmented acyl chains of oxPC protrude into the aqueous milieu [105] becoming physically accessible for cross-linking reactions with membrane proteins and downstream signalling with endothelial cells [[106], [107], [108], [109]]. OxPC are known oxidation-specific epitopes similar to danger-associated molecular patterns (DAMPs) involved in the inflammatory response [110,111] and hence the 3-fold increase in oxPC content in LDL observed in T2DM (Fig. 3B) contributes to its atherogenicity and increased inflammatory status.

### 4.4 Microbial (poly)phenol metabolites ameliorate the glucose-induced inflammatory response in cultured endothelial cells

In view of inflammatory-response induced by raised levels of glucose and circulating oxPC (Fig. 3) our results show that in spite of the reduced bioavailability of microbial (poly)phenol metabolites circulating in T2DM (Fig. 2A and Supplementary Fig. 3), treatment of glucose-challenged EC with sub-micromolar amounts of PCA, DHPPA and DHVL metabolites is able to mitigate the glucose-induced inflammatory response (Fig. 4) by reducing the release of pro-inflammatory IL-6 cytokine (Fig. 4A). The release of IL-1β was not evident after 6 h of incubation (Fig. 4B) when compared to IL-6 (Fig. 4A) suggesting a selective behaviour. The selective behaviour observed was unexpected since both IL-6 and IL-1 β cytokines are regulated by the NF-κB signalling pathway [112,113]. This could evidence that (poly)phenol metabolites exert their anti-inflammatory effect at a post-transcriptional level by preventing the post translational modifications (glycosylation) of IL-6 [114]. Our study stands out from others as we have focused on (poly)phenol metabolites in glucose-challenged EC at physiologically relevant concentrations (0.1–5 μM) and residence times (6 h), rather than dietary (poly)phenols [115,116]. Although, few works described the anti-inflammatory potential of colonic metabolites in endothelial cells these were cultured under normoglycemic conditions [21,40,45] while others have used longer (≥24 h) incubation periods [41,45] which far exceeded the residence times of (poly)phenol metabolites in circulation (Supplementary Fig. 5).

In summary, our work provides an improved understanding on the transport and bioavailability of circulating (poly)phenol metabolites in health and in T2DM. In addition, it also showcases the interplay of hypertriglyceridemic states with atherogenic LDL coexisting with oxidative HDL particles in T2DM. Further studies are mandatory to improve our understanding on the role of circulating (poly)phenol metabolites permeating the endothelial plasma membrane have on membrane proteins and their involvement in glucose transport across endothelial membrane.

## Funding information

This study was supported by the European Union through 10.13039/501100002924FEDER funds (NORTE-01-0145-FEDER-000052), the project AgriFood XXI - NORTE-01-0145-FEDER-000041, and supported by FCT/10.13039/501100006111MCTES (UIDB/50006/2020 and UID/BIM/0493/2020) through North Portugal Regional Operational Programme (Norte 2020). AR acknowledges funding from FCT – Fundação para a Ciência e a Tecnologia, I.P., within Norma Transitória - DL 57/2016/CP1346/CT0006. HKID gratefully acknowledges support from the Royal Society research grant RGS\R1\201135. JLS-Q and AP acknowledge support from FIS PI20/00334 and PI16/00471 from the 10.13039/501100004587Instituto de Salud Carlos III, Spanish 10.13039/100016145Ministry of Health (co-financed by the European Regional Development Fund). 10.13039/501100013941CIBERDEM (CB07/08/0016) is an Instituto de Salud Carlos III Project.

## Declaration of competing interest

The authors declare that they have no known competing financial interests or personal relationships that could have appeared to influence the work reported in this paper.

## Data Availability

Data will be made available on request.
