# Supplementary material for: Type 2 Diabetes mellitus alters the cargo of (poly)phenol metabolome and the oxidative status in circulating lipoproteins
Source: Redox Biol. 2022 Dec 5;59:102572. doi: 10.1016/j.redox.2022.102572 (PMC9762197; doi:10.1016/j.redox.2022.102572)
Supplement: Multimedia component 1 [file mmc1.docx]

**Supplementary Table 1.** Optimized electronic tune parameters used in ion-trap instrument (IT-MS, Themo Finningan) for the quantification of (poly)phenol metabolites in SPE extracts of isolated lipoproteins from normo- and hyperglycemic patients.

| **Parameters** | **Protocatechuic acid (PCA)** | **Hippuric acid (Hyp)** | **Di-hydroxyphenyl valerolactone (DHPV)** | **Di-hydroxyphenyl propanoic acid (DHPPA)** |
| --- | --- | --- | --- | --- |
| Capillary voltage (V) | -44 | -18 | -41 | -18 |
| Tube lens offset (V) | -60 | -60 | -60 | -60 |
| Multipole RF | 400 | 400 | 400 | 400 |
| Multipole 1 offset (V) | 9.0 | 9.0 | 0.75 | 9.0 |
| Multipole 2 offset (V) | 12.50 | 14.50 | 9.50 | 14.50 |
| Inter multipole (V) | 16 | 26 | 14 | 26 |
| Entrance lens (V) | 88 | 62 | 42 | 62 |
| Trap DC offset (V) | 10 | 10 | 10 | 10 |
