# Supplementary material for: Type 2 Diabetes mellitus alters the cargo of (poly)phenol metabolome and the oxidative status in circulating lipoproteins
Source: Redox Biol. 2022 Dec 5;59:102572. doi: 10.1016/j.redox.2022.102572 (PMC9762197; doi:10.1016/j.redox.2022.102572)
Supplement: Multimedia component 2 [file mmc2.docx]

**Supplementary Table 2.** Amount of microbial (poly)phenol metabolites quantified by targeted UPLC-LIT-(SIM)-MS in lipoprotein extracts. Data is expressed in pmol/mL (mean ± SD, n=3).

|  | **VLDL** | | | **LDL** | | | **HDL** | | |
| --- | --- | --- | --- | --- | --- | --- | --- | --- | --- |
|  | **NG** | **T2DM PC** | **T2DM GC** | **NG** | **T2DM PC** | **T2DM GC** | **NG** | **T2DM PC** | **T2DM GC** |
| **PCA metabolites** | 462±10 | ND | ND | 1943±102 | 5668±72 | 694±79 | 413±104 | 2064±138 | 300±14 |
| **DHPPA metabolites** | 374±57 | 402±82 | 377±14 | 345±28 | <LOQ | 420±78 | <LOQ | ND | ND |
| **DHPVL metabolites** | 14379±2487 | 13013±2629 | 13373±5668 | 13355±2349 | 12838±1798 | 2079±1402 | 21358±8445 | 12424±4181 | 20939±8760 |
| **Hippuric acid metabolites** | 237±25 | 141±25 | 209±24 | 286±24 | 113±8 | 111±4 | <LOQ | ND | ND |
