# Supplementary material for: Type 2 Diabetes mellitus alters the cargo of (poly)phenol metabolome and the oxidative status in circulating lipoproteins
Source: Redox Biol. 2022 Dec 5;59:102572. doi: 10.1016/j.redox.2022.102572 (PMC9762197; doi:10.1016/j.redox.2022.102572)
Supplement: Multimedia component 3 [file mmc3.docx]

Supplementary Material to

*Type 2 Diabetes Mellitus alters the cargo of (poly)phenol metabolome and the oxidative status in circulating lipoproteins*

Ana Reis^1,*^, Sara Rocha^1,a^, Irundika HK Dias^2^, Raquel Costa^3,4^, Raquel Soares^4,5^, José Luis Sánchez-Quesada^6^, Antonio Perez^7,8^, Victor de Freitas^1^

**Supplementary Figure 1.** Evaluation of extraction protocols (liquid-liquid extraction (LLE) and solid-phase extraction (SPE) using Oasis and hybrid-PL cartridges) in the extraction performance of phenolic compounds (μmol of gallic acid equivalents, GAE/mL) and phospholipids (μmol of inorganic phosphorous, PPi/mL) from plasma samples collected from normoglycemic fasting donors. Values depict mean ± SD (n=3). SPE protocols were done with 200μL according to manufacturer instructions. LLE was carried out on 200μL plasma by addition of 200μL HCl (0.5M):MeOH (1:1, v/v) vortexed for 10s every 2min for 15min, followed by extraction with 800μL ethyl acetate (99.8%, Panreac) and phase separation by centrifugation (5000rpm, 2min) at room temperature [Maiani et al., 1997]. Inset depicts the ratio of phenolic/phospholipid content.

**Supplementary Figure 2.** SDS-PAGE electrophoresis gel stained with colloidal Coomassie Blue of isolated lipoproteins by salt-gradient ultracentrifugation. Identification of proteins was based on the literature [Mahley et al., 1984].

**Supplementary Figure 3.** Estimated total (poly)phenol metabolites amount in lipoprotein extracts isolated from normo- and hyperglycemic donors quantified by targeted LC-MS. Values are expressed as mean±SD (n=3) normalized to ApoB-100 protein content (for VLDL and LDL) and normalized to ApoA-I protein content (for HDL). Significance levels ****p<0.0001 compared to normoglycemia.

**Supplementary Figure 4.** Evaluation of DHPV, PCA and DHPPA cytotoxicity by MTS assay on HMEC-1 cells exposed to normo- and hyperglycemia conditions (24h) prior to (poly)phenol metabolites treatment (6h). Data are expressed as mean percentage ± SD (n=3).

**Supplementary Figure 5.** Simulation of (poly)phenol metabolite circadian profile (μmol/L) in circulation in individuals following (poly)phenol-rich diets. The profile reflects 4-daily polyphenol-rich meals (breakfast, lunch, afternoon snack and dinner). The simulation (adapted from [Rocha et al., 2021] showing the profile of (poly)phenol conjugates (dotted line, corresponding to methyl-, sulphate- and glucuronic- derived metabolites) and of (poly)phenol microbial metabolites (dashed line, ring-fission metabolites), takes into consideration daylight and sleeping periods.

**Reagents**

Reagents used for spectrophotometric characterization were of highest purity commercially available. Sodium carbonate (Na_2_CO_3_), Folin-Ciocalteu reagent, sodium nitrate (NaNO_2_), 2,2’-Diphenyl-1-picrylhydrazyl (DPPH), trolox (6-hydroxy-2,5,7,8-tetramethylchroman-2-carboxylic acid), sodium acetate (CH_3_COONa); ferric chloride (FeCl_3_); 2,4,6-tris(2-pyridyl)-S-triazine (TPTZ), sodium molibdate (NaMoO_4_.H_2_O), and ascorbic acid were purchased from Sigma Aldrich. Aluminium chlorite hexahydrate (AlCl_3_.6H_2_O) and catechin (purity ≥98%) were purchased from Fluka; phosphate-buffered saline (PBS) buffer, sodium hydroxide (NaOH) and ferrous sulphate (FeSO_4_) were purchased from PanReac AppliChem. Gallic acid (purity 97.5-102.5%) and (poly)phenol metabolite standards used 3-(2,4-Dihydroxyphenyl)propanoic acid (DHPPA, purity >95%), hippuric acid (Hyp, purity >98%) from Sigma-Aldrich; 3-(3’,4’-dihydroxyphenyl)-γ-valerolactone (DHPV, purity >98%) and protocatechuic acid (PCA, purity >99%) were purchased from HWI Group (Mannheim, Germany).

Solvents used for extraction and LC-MS analysis include formic acid (LC-MS grade, Thermo Scientific) acetonitrile (Chem-Lab) and ultrapure water from a Milli-Q system (Rephile). t-MBE (Riedel-de-Haën) Cell culture reagents including RPMI 1640 medium and fetal bovine serum (FBS), penicillin/streptomycin were purchased from Invitrogen Life Technologies, Pierce BCA Protein Assay Kit from Thermo Scientific, sodium bicarbonate, 4-(2-hydroxyethyl)-1-piperazineethanesulfonic acid buffer (HEPES), endothelial growth factor (EGF), hydrocortisone (purity >98%), and ELISA kits were purchased from Sigma-Aldrich.

**Study Group Information and Lipoprotein isolation**

Isolated lipoprotein samples (HDL, LDL and VLDL) from diabetic (n=15) and control group (n=15) were prepared from pooled plasma collected in EDTA tubes from normo- and diabetic donors recruited at the Hospital de la Santa Creu I Sant Pau (Barcelona, Spain). Normoglycemic and normolipidemic donors (Control group, HbA1c < 6%) consisted of 7 males and 8 females with an average age of 49 ± 8 years and body mass index (BMI) of 26.3 ± 1.7 kg/m^2^. Diabetic donors (Hyperglycemia poor control (PC), HbA_1c_ > 8.5%) consisted of 7 males and 8 females with an average age of 57 ± 9 years, and BMI of 27.1 ± 3.0 kg/m^2^. The same diabetic patients underwent individualized hypoglycemic therapy (metformin, insulin, diet, and/or moderate exercise) to achieve good glycemia control (Hyperglycemia good control (GC), HbA_1c_ < 6.5%). In addition to hypoglycemic therapies, 35% of diabetic patients were treated with hypolipemic drugs (statins) and 15% with antihypertensive drugs. Patients with acute or chronic infections, clinically assessed cardiac disease, active inflammatory disease, treatment with anti-inflammatory drugs or C-reactive protein (CRP)>20 mg/L were excluded.

The lipid and apolipoprotein composition of isolated purified (VLDL (1.006–1.019 g/mL), LDL (1.019–1.063 g/mL), and HDL (1.063–1.210 g/mL) was determined by measuring the content of cholesterol, triglycerides, apoB, apoA-I (Roche Diagnostics), phospholipids and free cholesterol (Wako Pure Chemical, Osaka, Japan) in a Cobas 6000/c501autoanalyzer and data expressed as percentage of lipoprotein mass. Anthropometric characteristics, lipid profile, HbA_1c_ and CRP levels of all subjects studied are shown in **Table 1**.

**Purity of lipoprotein samples by SDS gel electrophoresis**

Purity of isolated lipoproteins was confirmed by SDS gel electrophoresis loaded on a gradient polyacrylamide gel (4-15%, BioRad, Hempstead, UK) run for 1 hr at 120V in a Mini-Protean II electrophoresis tank. Protein in samples were identified against protein markers (10-250 kDa, BioRad, Hempstead, UK). Gels were fixed with homemade colloidal Coomassie Blue solution with constant stirring at room temperature in an orbital shaker.

**Quantification of (poly)phenol metabolites in lipoprotein extracts by reverse-phase liquid chromatography-mass spectrometry (LC-MS)**

Dried lipoprotein extracts were resuspended in 100 μL of 10% ACN (v/v) and the (poly)phenol metabolites separated in a reverse-phase Hypersil GOLD™ VANQUISH™ C18 UHPLC Colum (150 mm x 2.1 mm, 1.9 μm) coupled to a Finnigan LCQ DECA XP MAX quadrupole ion trap equipped with an atmospheric pressure ionization (API) source, using electrospray ionization (ESI) interface. Mobile phases consisted of ultrapure water with 1% formic acid (mobile phase A) and ACN containing 1% formic acid (mobile phase B). The volume of sample injected was 25 μL with a flow rate of 150 μL/min with the autosampler at 20ºC and following the gradient: 2%B from 0 to 3 min, 20% B from 3 to 12 min, 20-95% from 12 to 17 min, 95% B 17-25 min, 95-2% B 25-29 min, 2%B 29-35 min. Detection of standard and (poly)phenol metabolites was performed in the negative mode using selected ion mode (SIM) with an ionization voltage of -5 kV, an ion source temperature of 325^o^C and sheath gas flow 40 (arbitrary units).

Optimization of MS detection parameters was achieved using standard compound solutions of DHPV, PCA, DHPP and hippuric acid (poly)phenol metabolites infused directly to the ESI source through an integrated syringe pump (Harvard apparatus) at a flow rate of 18 μL/min. Sample vials were placed in the autosampler at 10ºC and analysis of (poly)phenol metabolites in lipoprotein extracts was achieved by injection of 25μL in triplicate with blank runs in between each three injections. The LC-MS chromatograms were then analysed by XCalibur software (version 2.2). Analyte concentration was estimated using the area under the curve (AUC) smoothed with Savitsky-Golay (7) plotted in calibration curves built using optimised tuning parameters (**Supplementary Table 1**) for the commercially available standards (DHPV, PCA, DHPP and hippuric acid). The screened sulphate and glucuronic acid conjugates of DHPV, PCA, DHPP and hippuric acid microbial metabolites were monitored at the theoretical *m/z* value and their concentrations estimated against the corresponding metabolite calibration curve obtained. The content of individual (poly)phenol metabolites was expressed as pmol/mg protein.

**Cell culture of human microvascular endothelial cells**

Human microvascular endothelial cells (HMEC-1, ATCC) were cultured in RPMI 1640 medium supplemented with 10% FBS, 1% penicillin/streptomycin, 1.176 g/L sodium bicarbonate, 4.76 g/L HEPES, 10 g/mL EGF and 1 mg/L hydrocortisone (purity >98%) and maintained at 37ºC in a humidified 5% CO_2_ atmosphere. All experiments were performed between cell passages 6 and 11. Treatment of HMEC-1 cells was done in serum-free cell medium with 5.5 mM glucose, to mimic the normoglycemic condition, or 30 mM glucose concentration, to mimic diabetic condition for 24h followed by the addition of the (poly)phenol metabolites (0.1-5 μM) to the medium and left to incubate for 6h. This procedure was done in triplicate. Cell supernatants were collected and stored at -20ºC until required.

**Cell viability assay**

Cells were sub-cultured in 96-well plates at 2x10^5^ cells/mL of medium. HMEC-1 cells were treated in triplicate for 24h in serum-free conditions with 5.5 mM or 30 mM glucose concentration. Next, the medium was replaced by fresh medium with 5.5 mM or 30 mM glucose where it was added the (poly)phenol metabolites. The control used was 0.1% ethanol. Afterwards, the medium with the (poly)phenol metabolites was removed and substituted with medium with glucose and tetrazolium salt (MTS). After 1h of incubation in the dark, the absorbance at λ=492 nm was measured and results expressed as percentage change relative to control (medium with 5.5 mM glucose).

**Enzyme-linked immunosorbent assay for inflammatory markers IL-6 and IL-1β**

Quantification of cytokines (IL-6 and IL-1β) in HMEC supernatant was achieved by ELISA protocols (Sigma-Aldrich, Germany) according to the manufacturer’s instructions. HMEC-1 were seeded and allowed to grow to 24x10^4^ cells/well in 24-well plates and treated as described in section 2.8. The control used was 0.1% (v/v) ethanol. The results are expressed as pg/mL.

Reference List

G. Maiani, M. Serafini, M. Salucci, E. Azzini, A. Ferro-Luzzi, Application of a new high-performance liquid chromatographic method for measuring selected polyphenols in human plasma, Journal of Chromatography B: Biomedical Applications. 692 (1997) 311–317.

R.W. Mahley, T.L. Innerarity, S.C. Rall Jr., K.H. Weisgraber, Plasma lipoproteins: apolipoprotein structure and function, J Lipid Res. 25 (1984) 1277–1294.

S. Rocha, O. Oskolkova, V. de Freitas, A. Reis, (Poly)phenol-Rich Diets in the Management of Endothelial Dysfunction in Diabetes Mellitus: Biological Properties in Cultured Endothelial Cells, Molecular Nutrition and Food Research. 65 (2021) 1–11.
